# Candy Closure Technique for Chronic Open Infective Lateral Malleolus Bursitis

**DOI:** 10.1155/2019/5490139

**Published:** 2019-03-17

**Authors:** Jin Su Kim, Woo Jong Kim, Ki Won Young, Kyu Hwan Bae, Han Hoon Kim, Hong Seop Lee

**Affiliations:** ^1^Department of Orthopaedic Surgery, CM General Hospital, 13 Yeongdeungpo-ro 36-gil, 07301 Seoul, Republic of Korea; ^2^Department of Orthopaedic Surgery, Soonchunhyang University Hospital Cheonan, 31 Suncheonhyang 6-gil, Dongam-gu, 31151 Cheonan, Republic of Korea; ^3^Department of Foot and Ankle Surgery, Eulji Medical Center, Eulji University, 68 Hangeulbiseok-ro, Nowoungu, 01830 Seoul, Republic of Korea

## Abstract

The aim of this study was to report the effectiveness of the Candy closure technique as a treatment for chronic open infective lateral malleolus bursitis. From June 2014 to March 2018, we performed the Candy closure technique as a treatment for chronic open infective lateral malleolus bursitis in nine patients without secondary operation. We first performed infectious tissue debridement to control infection, and if primary closure was not possible, we performed the Candy closure technique for small wounds. The duration of the wound prior to surgery varied from 4 weeks to 2 years. Seven cases were due to infection on the bursa and two cases were ulcer-type bursitis. All the wounds were small (average, 3.80 cm^2^; range, 2.25-4 cm^2^) and circular. Seven wounds showed complete healing at 4 weeks after surgery, one wound showed complete healing at 8 weeks after surgery, and one wound with infected state was lost to missing follow-up. Of the seven wounds that showed complete healing, one wound recurred 6 months after surgery. The Candy closure technique is a simple method for ensuring healing and coverage of chronic open lateral malleolus bursitis, especially for small wounds with dead space.

## 1. Introduction

A bursa is a cyst lined with synovial cells, and its function is to reduce friction over areas of high pressure. There are two types of bursae: anatomical and adventitious. Both the anatomical and adventitious bursae may become pathological under chronic pressure and may develop thick walls, distension, inflammation, and even suppuration [1, 2]. Lateral malleolar bursitis develops as a result of chronic pressure on the lateral malleolar region of the ankle. This is particularly problematic in skaters and also in Asians who might sit cross-legged [3]. The first line of treatment for lateral malleolar bursitis includes lifestyle changes, avoidance of cross-legged sitting, aspiration, or use of compressive wrap [4]. However, the recurrence rate is high [5], and infection may occur during treatment. When infection develops, the bone may become exposed because of the thin overlying soft tissue [6]. It becomes intractable in compromised patients with abnormal sensation or circulatory disturbance in the legs. After debridement of the infective lateral malleolus bursitis, a circular skin defect is formed in the lateral malleolus area. A simple suture of a circular defect wound causes a lot of tension and a large dog ear to remain. If simple suture is not possible due to soft tissue defect, a skin graft, pedicled tissue transfer, and free tissue transfer can be used for covering the exposed bone [5–8]. Local flaps may be used for reconstruction of soft tissue defects in foot and ankle joints, but the area of available mobile skin is limited [8]. Skin graft or flap surgery may have donor site problem and necessitate general or spinal anesthesia, which may be dangerous to elderly patients with chronic illness.

We performed the Candy closure technique as a treatment for chronic open infective lateral malleolus bursitis in nine cases without secondary operation. We hypothesized that the technique would show good clinical results. This report presents the clinical results and efficacy of the procedure.

## 2. Materials and Methods

This study was reviewed and approved by the Institutional Review Board of Eulji Medical Center (Approval no. 2018-01-005). The requirement for informed consent was waived because of the retrospective nature of the study. From June 2014 to March 2018, we performed the Candy closure technique as a treatment for chronic open infective lateral malleolus bursitis in nine patients. We first performed infectious tissue debridement to control infection, and if primary closure was not possible, we performed the Candy closure technique for small wounds. Eight of these patients were male, and the lesion was on the left side in 4 patients. The average age was 62.25 (range, 41–74) years. We retrospectively reviewed the patients' chart, including their medical history, wound duration, vascular evaluation, wound cause, preoperative management, wound size, culture, surgery time (minutes), healing time (weeks), follow-up time (months), last wound status, and surgical complications and recovery time of ankle joint range of motion (ROM).

### 2.1. Surgical Technique

**Figure 1:**
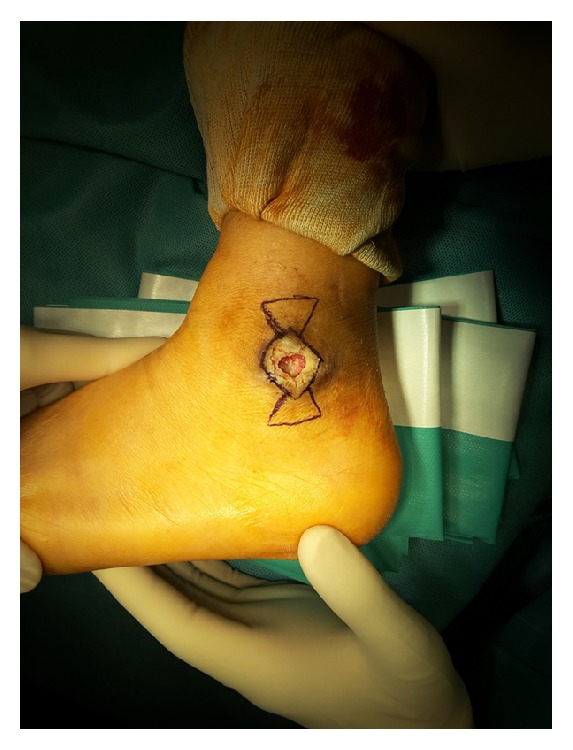
Incision marking was performed between the angiosome of the anterior tibial artery and the peroneal artery. A rectangular flap with the wound defect was made and thena triangular flap was drawn above and below the rectangular flap.

**Figure 2:**
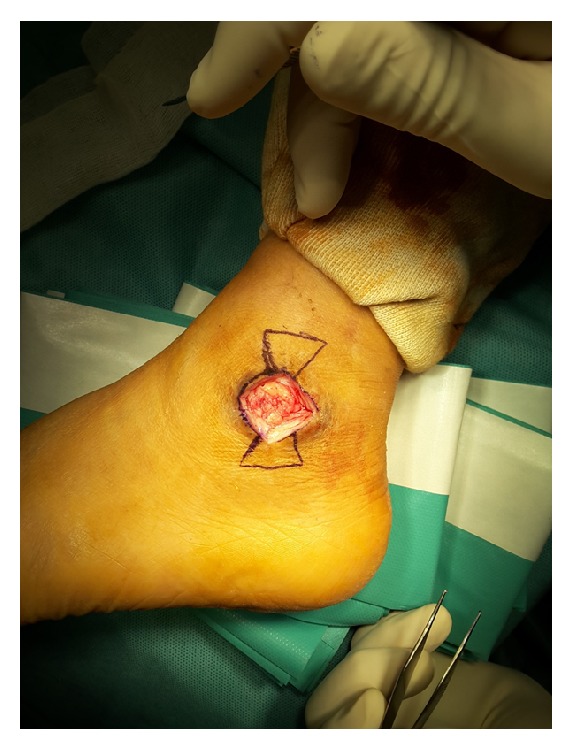
The rectangular flap containing a wound defect was excised.

**Figure 3:**
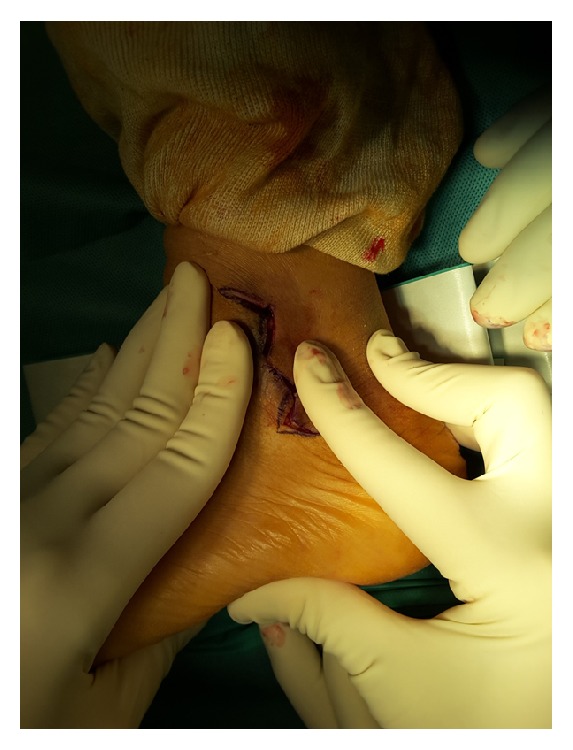
The flap on the anterior part was pushed up and the flap on the posterior part was pushed down to make a Zigzag wound.

**Figure 4:**
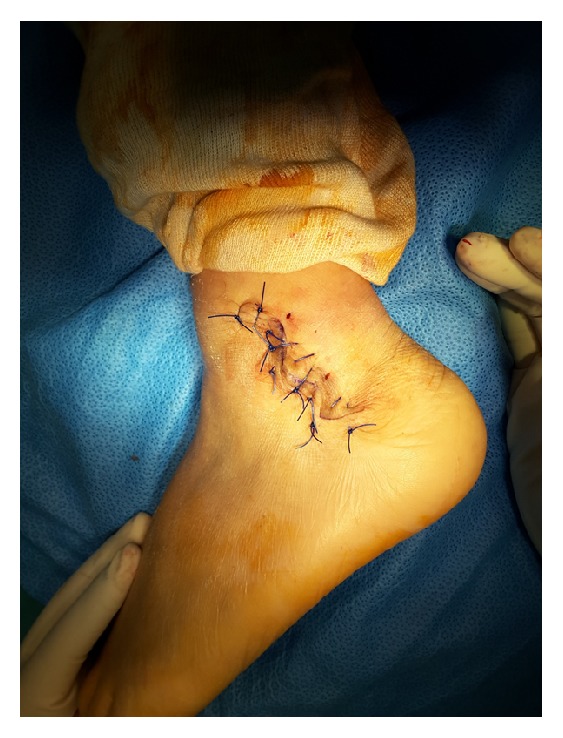
The flaps are approximated and sutured with Nylon 1.0.

**Figure 5:**
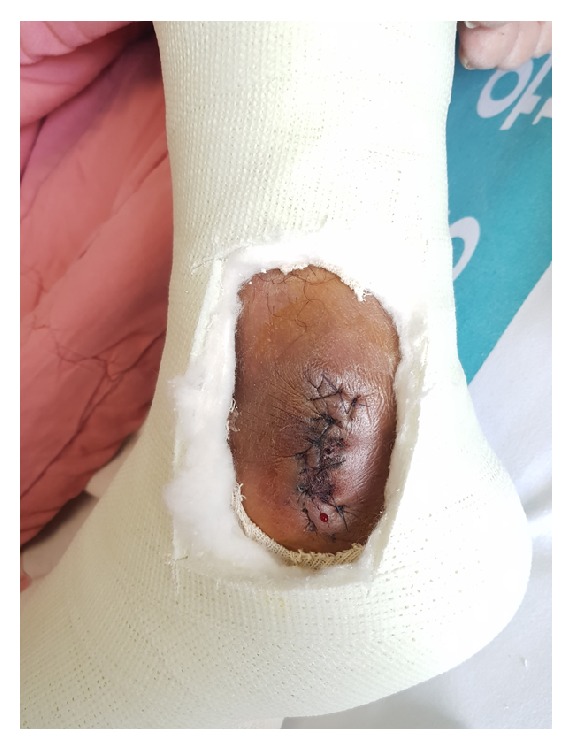
The ankle was placed in a short leg cast immediately after surgery, which was maintained for 4 weeks.

All surgeries were performed after the infected tissue was completely removed. Under popliteal block, we used the Esmarch tourniquet above the ankle level. Incision marking was performed between the angiosome of the anterior tibial artery and the peroneal artery [9]. A rectangular flap with the wound defect was made; then, a triangular flap was drawn above and below the rectangular flap (Figure 1). The rectangular flap and two triangular flaps on each side of the wound defect were excised simultaneously (Figure 2). The layer of the flap excised was full thickness skin flap that including entire dermis layer. The flap on the anterior part was pushed up and the flap on the posterior part was pushed down to make a zigzag wound (Figure 3). The flaps are approximated and sutured with Nylon 1.0 (Figure 4). The ankle was placed in a short leg cast immediately after surgery; it was maintained for 4 weeks and removed during suture removal (Figure 5). The patient was instructed to perform an active and passive ankle joint ROM.

## 3. Results

**Figure 6:**
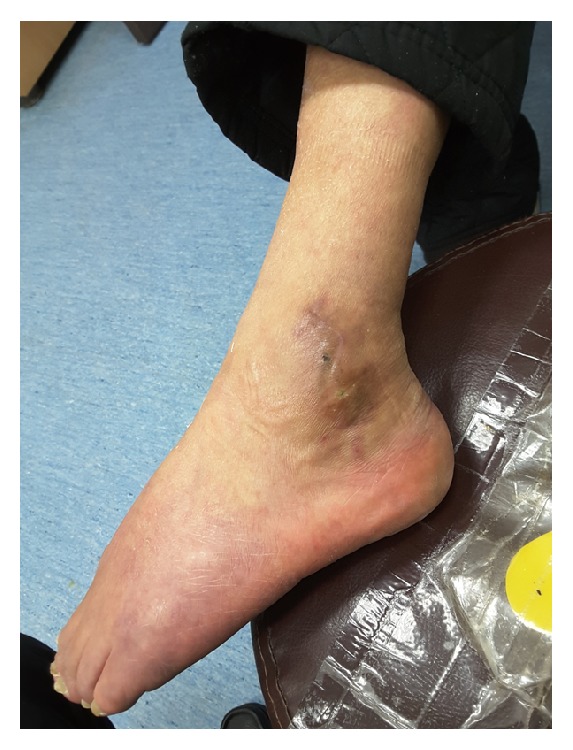
Complete wound healing maintained at 8 weeks after surgery.

**Figure 7:**
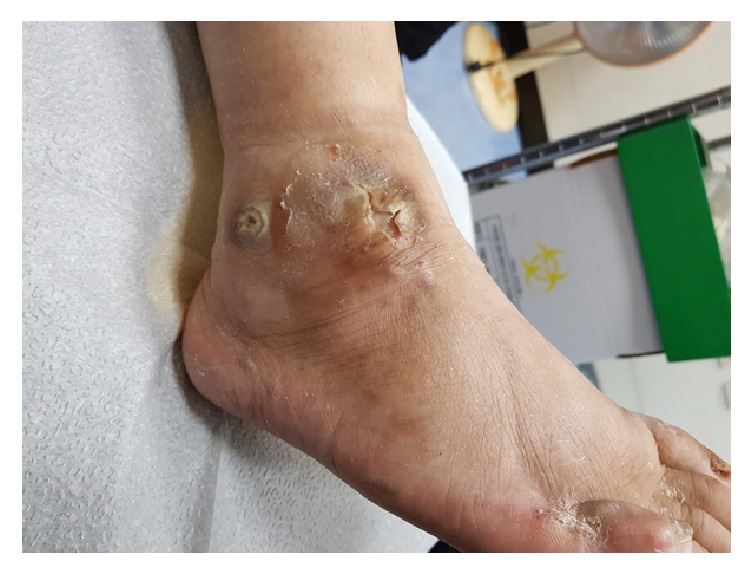
In patient 3, the wound recurred at 6 months postoperatively.

All patients had significant medical history. Eight patients had diabetes mellitus, five patients had hypertension, two patients had a history of cerebrovascular accident, three patients were receiving hemodialysis, and one patient was paraplegic. The duration of the wound before surgery varied from 4 weeks to 2 years. Seven cases were due to infection on the bursa, and two cases were ulcer-type bursitis (one was a pressure ulcer due to sensory insufficiency secondary to paraplegia and the other one was due to prolonged pressure after contralateral below knee amputation). Seven wounds after suppurative infection on bursa were debrided several times. All the wounds were small (average, 3.80 cm^2^; range, 2.25-4 cm^2^) and circular. At the time of surgery, various bacteria were cultured in seven wounds, but not in two wounds. Seven wounds showed complete healing at 4 weeks after surgery, one wound showed complete healing at 8 weeks after surgery (Figure 6), and one wound with infected state was lost to missing follow-up. Of the seven wounds that healed completely, one wound recurred 6 months after surgery (Figure 7). Average recovery time of ankle joint ROM was 4.25 weeks (range; 3-6 weeks). Detailed patient data are described in Table 1.

## 4. Discussion

The lateral malleolus is an important constituent of the ankle joint. However, it has no muscle coverage [10], and only a thin, friable, and mobile envelope of skin provides protection for the important structure [11]. Moreover, there is no major blood flow in the area [12]. These features make wound healing around the lateral malleolus problematic [5]. The area around the lateral malleolus is weaker because of the high pressure it sustains. This high pressure is usually generated by the cross-legged position common in specific regions or by general weakness induced by external rotation of the hip [6]. Because lateral malleolar bursitis recurrence is common, several studies on treatment methods aiming to address bursitis without recurrence have been reported such as indwelling silk suture [13], endoscopic bursectomy [4], and sclerotherapy [14]. However, these methods cannot be used in cases with open wounds on the lateral malleolus.

Repeated debridement to control infection results in soft tissue defects with dead space. In this case, simple suture is impossible and reconstructive surgery is necessary. Reconstruction of soft tissue defect in the foot and ankle joints has long been a challenge, as the skin around this area does not have good blood circulation and has low elasticity and mobility [15, 16]. For such reconstruction, several types of flaps including local flaps, regional flaps, and free flaps have been used. However, flap surgery has disadvantages, as it leaves a big scar and needs additional graft surgery [6]. If the flap surgery is successful, there is a possibility of pressure ulcer when shoes are worn because the flap tissue is thick. It takes a long time for the granulation tissue to fill the dead space in order to perform split thickness skin graft.

The Candy closure technique used in this study was simple and was performed under only popliteal anesthesia; thus, there were no complications related to graft and anesthesia. Furthermore, no secondary surgery was necessary. However, this method can only be used on a small wound, and it requires a short leg cast immobilization for 4 weeks after surgery. Patients with diabetic neuropathy may develop pressure ulcers due to the short leg cast. A scar contracture remains after surgery because the surrounding tissue is resected, which may lead to discomfort in joint movement.

The limitations of this study include the retrospective study design and small number of cases. Moreover, there was no comparison with a group treated with another method. We did not evaluate the vascular supply before surgery. If the blood circulation around the wound is insufficient, other treatments should be considered. A randomized study is needed to compare the results of flap surgery and the Candy closure technique.

## 5. Conclusions

The Candy closure technique ensures healing and coverage of chronic open lateral malleolus bursitis, especially for small wounds with dead space. With its simplicity, the technique can be used reliably in the treatment of chronic open lateral malleolus bursitis.

## Figures and Tables

**Table 1:** This table shows detailed patient data.

No.	Sex	Age	Wound duration	Cause	Past medical history	Pre-op management	wound size	culture	Op time	Healing time(weeks)	Follow-up(months)	Recovery time of ankle joint ROM	Last status
1	M	41	19 weeks	Infective lateral malleolar bursitis	HBV carrier, DM CKD	Debridement & Irrigation	2*∗*2cm	Corynebacterium minutissimum	30min	4	12	4	healing

2	M	57	10 weeks	Infective lateral malleolar bursitis	DM	Debridement & Irrigation	2*∗*2cm	No growth	30min	4	12	3	healing

3	F	57	7 weeks	Ulcer type bursitis	DM, CKD, MI	Curettage	1.5*∗*2cm	Proteus mirabilis	1hr	4	6	5	Recurrence

4	M	72	4 weeks	Infective lateral malleolar bursitis	DM, HTN, CVA	Debridement & Irrigation	1.5*∗*2cm	Pseudomonas aeruginosa	1hr	4	48	4	healing

5	M	72	4 weeks	Infective lateral malleolar bursitis	DM, HTN, CVA	Debridement & Irrigation	2*∗*2cm	Pseudomonas aeruginosa	30min	4	48	4	healing

6	M	73	2 years	Infective lateral malleolar bursitis	DM, HTN	Debridement & Irrigation	2*∗*2cm	Coagulase negative staphylococcus	1hr	8	48	6	healing

7	M	68	3 years	Infective lateral malleolar bursitis	DM, HTN, CKD	Debridement & Irrigation	2*∗*2cm	MSSA	30min	4	40	4	healing

8	M	58	1 year	Ulcer type bursitis	Paraplegia	Curettage	1.5*∗*2cm	No growth	1hr	4	12	4	healing

9	M	74	1 year	Infective lateral malleolar bursitis	DM, HTN	Debridement & Irrigation	1.5*∗*1.5cm	Enterococcus faecalis-Group D	1hr	Not healed	4	Follow up loss	Follow up loss

Op: operation, CVA: cerebral vascular accident, DM: diabetes mellitus, HTN: hypertension, CKD: chronic renal disease, HBV: hepatitis B virus, MI: myocardial infarction, ATA: anterior tibial artery, PTA: posterior tibial artery, ASO; atherosclerotic obliterans, MSSA: methicillin sensitive *Staphylococcus aureus*, and ROM: range of motion.

## Data Availability

The data used to support the findings of this study are included within the article.
